# Optimal timing for the first cystoscopic follow-up using time-to-treatment initiation analysis of oncologic outcomes in primary non-muscle invasive bladder cancer

**DOI:** 10.1038/s41598-024-58809-x

**Published:** 2024-04-10

**Authors:** Jeong-Soo Kim, Jooyoung Lee, Tuan Thanh Nguyen, Se Young Choi

**Affiliations:** 1 Department of Applied Statistics, Chung-Ang University, Seoul, South Korea; 2 Department of Urology, Cho Ray Hospital, University of Medicine and Pharmacy at Ho Chi Minh City, Ho Chi Minh City, Vietnam; 3 Department of Urology, Chung-Ang University Hospital, Chung-Ang University College of Medicine, 102, Heukseok-ro, Dongjak-gu, Seoul, 06973 South Korea

**Keywords:** Cystoscopy, Follow-up, Non-muscle invasive bladder cancer, Restricted cubic spline function, Time to treatment initiation, Health care, Oncology, Urology

## Abstract

Various guidelines recommend the first follow-up cystoscopy at 3 months; however, no data exist on the optimal timing for initial follow-up cystoscopy. We tried to provide evidence on the timing of the first cystoscopy after the initial transurethral resection of bladder tumor (TUR-BT) for patients with non-muscle invasive bladder cancer (NMIBC) using big data. This was a retrospective National Health Insurance Service database analysis. The following outcomes were considered: recurrence, progression, cancer-specific mortality, and all-cause mortality. Exposure was the time-to-treatment initiation (TTI), a continuous variable representing the time to the first cystoscopy from the first TUR-BT within 1 year. Additionally, we categorized TTI (TTIc) into five levels: < 2, 2–4, 4–6, 6–8, and 8–12 months. A landmark time of 1 year after the initial TUR-BT was described to address immortal-time bias. We identified the optimal time for the first cystoscopy using Cox regression models with and without restricted cubic splines (RCS) for TTI and TTIc, respectively. Among 26,660 patients, 16,880 (63.3%) underwent cystoscopy within 2–4 months. A U-shaped trend of the lowest risks at TTI was observed in the 2–4 months group for progression, cancer-specific mortality, and all-cause mortality. TTI within 0–2 months had a higher risk of progression (aHR 1.36; 95% confidence intervals [CI] 1.15–1.60; p < 0.001) and cancer-specific mortality (aHR 1.29; 95% CI 1.05–1.58; p = 0.010). Similarly, TTI within 8–12 months had a higher risk of progression (aHR 2.09; 95% CI 1.67–2.63; p < 0.001) and cancer-specific mortality (aHR 1.96; 95% CI 1.48–2.60; p < 0.001). Based on the RCS models, the risks of progression, cancer-specific mortality, and all-cause mortality were lowest at TTI of 4 months. The timing of the first cystoscopy follow-up was associated with oncologic prognosis. In our model, undergoing cystoscopy at 4 months has shown the best outcomes in clinical course. Therefore, patients who do not receive cystoscopy at approximately 4 months for any reason need more careful follow-up to predict a poor clinical course.

## Introduction

Bladder cancer ranks second among the most prevalent genitourinary tract malignancies in males and tenth globally in both sexes^1^. Non-muscle invasive bladder cancer (NMIBC) accounts for approximately 75% of cases and is restricted to the mucosa or submucosa^2^. Despite its higher prevalence and recurrence rates, NMIBC has a better cancer-specific mortality rate than muscle-invasive bladder cancer^3^. The recurrence rate is > 50% at the first cystoscopy, traditionally performed 3 months after transurethral resection of bladder tumor (TUR-BT)^4^. Patients and physicians are displeased with the high recurrence rate and repeated cystoscopies after TUR-BT. Cystoscopy demonstrates high sensitivity and specificity in detecting bladder cancer. Thus, other currently available tests, such as urinary markers and cytology, cannot replace cystoscopy in most situations^5^.

Various guidelines recommend the first follow-up cystoscopy at 3 months; however, data that define the optimal timing for initial follow-up cystoscopy are unavailable^6,7^. Previously, the initial follow-up of cystoscopy at 3 months was traditional^8,9^. The initial cystoscopy after TUR-BT at 3 months is an important prognostic indicator for recurrence and progression^8,9^. Therefore, guidelines strongly recommend cystoscopy at 3 months. However, the reason for this is unknown. A randomized controlled trial that compared 6- and 3-month follow-up protocols for NMIBC (Ta)^10^ observed that the small sample size may have limited its capacity to detect remarkable differences; however, no significant differences existed in recurrence or progression^10^. In practice, 84% of patients undergo their first cystoscopy 3–4 months after TUR-BT^11^. The timing of the first follow-up cystoscopy is critical; hence, a uniform protocol is needed. Therefore, we aimed to provide evidence on the timing of the first cystoscopy after TUR-BT and assess the oncologic outcomes in NMIBC.

## Materials and methods

### Data source

Data was obtained from the National Health Claims Database released by the National Health Insurance Service (NHIS). This public universal healthcare system provides comprehensive medical care coverage to 99% of Koreans (> 50 million individuals). The Institutional Review Board of Chung-Ang University Hospital approved this study, and the need for informed consent was waived. The study adhered to the guidelines of the Declaration of Helsinki. This study protocol was approved by the ethical review board at Chung-Ang University Hospital (IRB no. 1908-014-16279).

### Study population

A total of 67,796 newly diagnosed patients with NMIBC between 2004 and 2016 who underwent TUR-BT were identified as those with KCD-8 code C67 and surgical code R3512. This cohort did not include the D codes of D090 (Carcinoma in situ of bladder) or D414 (Neoplasm of uncertain behavior of bladder).

**Figure 1:**
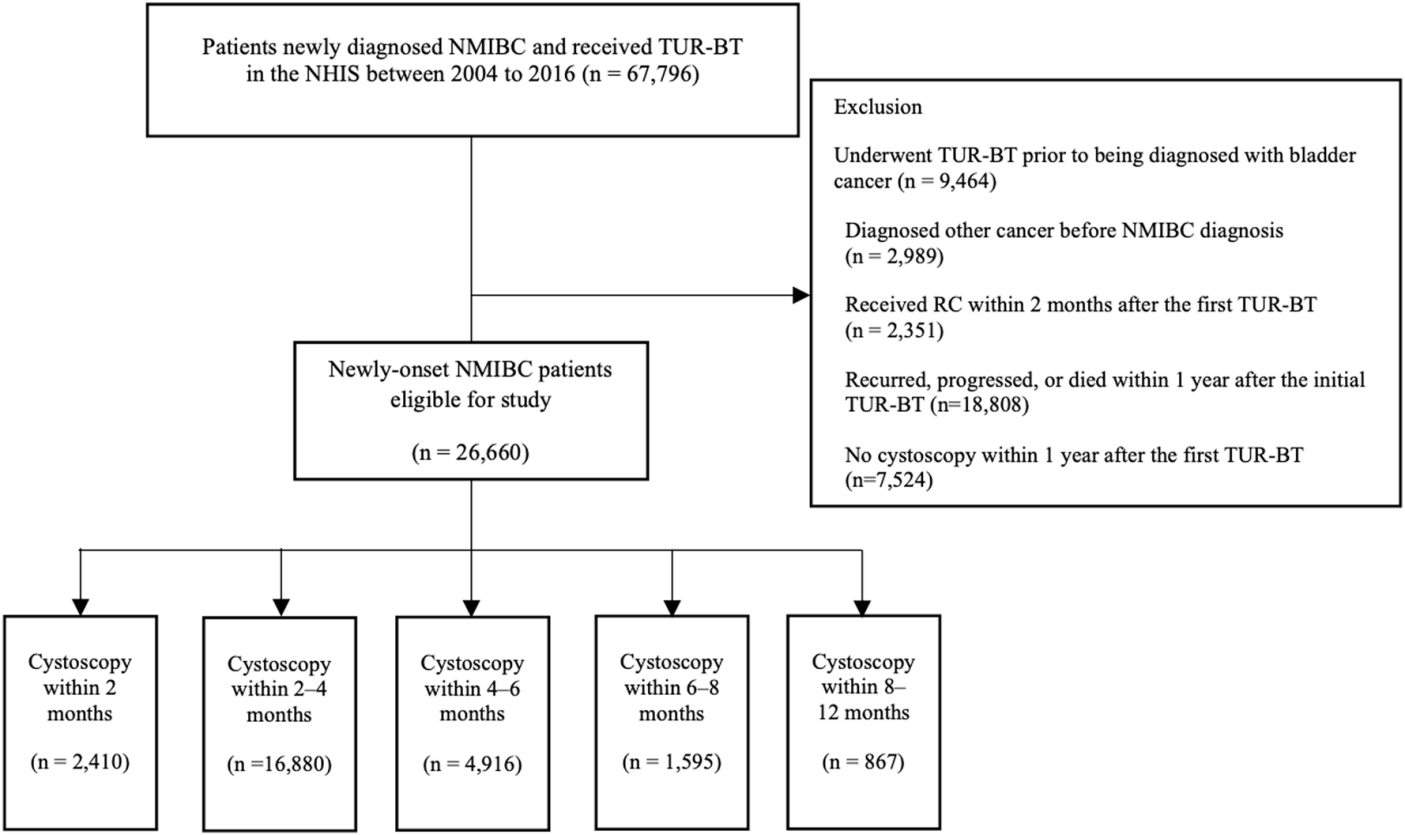
Flow chart of the study design. *NMIBC* non-muscle invasive bladder cancer, *TUR-BT* transurethral resection of bladder tumor, *NHIS* National Health Insurance Service, *RC* radical cystectomy.

Patients who underwent TUR-BT prior to being diagnosed with bladder cancer (n = 9464), any other cancer before NMIBC (n = 2989), or underwent radical cystectomy (RC) within 2 months after the first TUR-BT (n = 2351) were excluded. To prevent immortal-time bias, we set one year after the first TUR-BT as the landmark time and excluded patients who experienced recurrence or progression or died before the landmark time (n = 18,808). Patients who had never undergone cystoscopy within one year of the first TUR-BT were also excluded (n = 7524). In total, 26,660 patients were eligible for this study (Fig. 1).

### Outcomes and variables

Three outcomes were considered in this study: (1) recurrence—patients who underwent TUR-BT or RC; (2) progression—patients who underwent chemotherapy or RC; (3) cancer-specific mortality—patients who died due to cancer after progression; and (4) all-cause mortality—patients who died. The index date was the first TUR-BT date. Participants were observed until death, loss to follow-up, or December 31, 2018, whichever came first.

Exposure was the time-to-treatment initiation (TTI), a continuous variable defined as the time to the first cystoscopy within a year of the initial TUR-BT. In addition, TTI was categorized (TTIc) into five levels: < 2, 2–4, 4–6, 6–8, and > 8 months. The following were the covariates: sex, age at NMIBC diagnosis, year of NMIBC diagnosis, Charlson Comorbidity Index (CCI) score based on comorbidities before NMIBC diagnosis, repeated TUR-BT (TUR-BTs follow-up status in 2 months after the initial TUR-BT), Bacillus Calmette-Guérin (BCG) usage, and upper tract urothelial cancer (UTUC) occurrence before NMIBC diagnosis. Age at NMIBC diagnosis year was categorized into $<$ 55, 55–64, 65–74, and $\ge$ 75. CCI scores—a measure of comorbidities—were categorized into three groups: 0–1, 2–3, and $\ge$ 4. NMIBC diagnosis years were divided into 2004–2008, 2009–2012, and 2013–2016.

### Statistical analyses

Demographic and clinical characteristics were expressed as means $\pm$ standard deviations (SD), median [interquartile range (IQR)], or numbers with percentages. The chi-square test was used to compare categorical variables. Kaplan–Meier curves were generated to compare the differences in survival probabilities among the TTI groups, whereas the log-rank test compared survival probabilities. Multivariable Cox proportional hazard models were used to examine the association between TTIc and the risks of recurrence, progression, cancer-specific mortality, and all-cause mortality, while adjusting for age, sex, diagnosis year, CCI, repeated TUR-BT, BCG usage, and occurrence of UTUC. Furthermore, multivariable Cox regression models with restricted cubic splines were used to investigate the nonlinear relationships between the risk of each outcome and TTI. Seven knots were placed at the 5th, 10th, 25th, 50th, 75th, 90th, and 95th percentiles of the observed TTI distribution, and the reference value for the hazard ratio was a TTI of 3 months. Subgroup analyses were conducted according to age ($<$ 55, 55–64, 65–74, and $\ge$ 75), sex, CCI score (0–1, 2–3, and $\ge$ 4), repeated TUR-BT, and BCG usage.

Data were statistically analyzed using the SAS version.7.0 (SAS Institute Inc., Cary, NC, USA) and R software, version 4.0.1 (R Foundation for Statistical Computing, Vienna, Austria). For all analyses, statistical significance was set at p < 0.05.

## Results

### Baseline characteristics of patients

**Figure 2:**
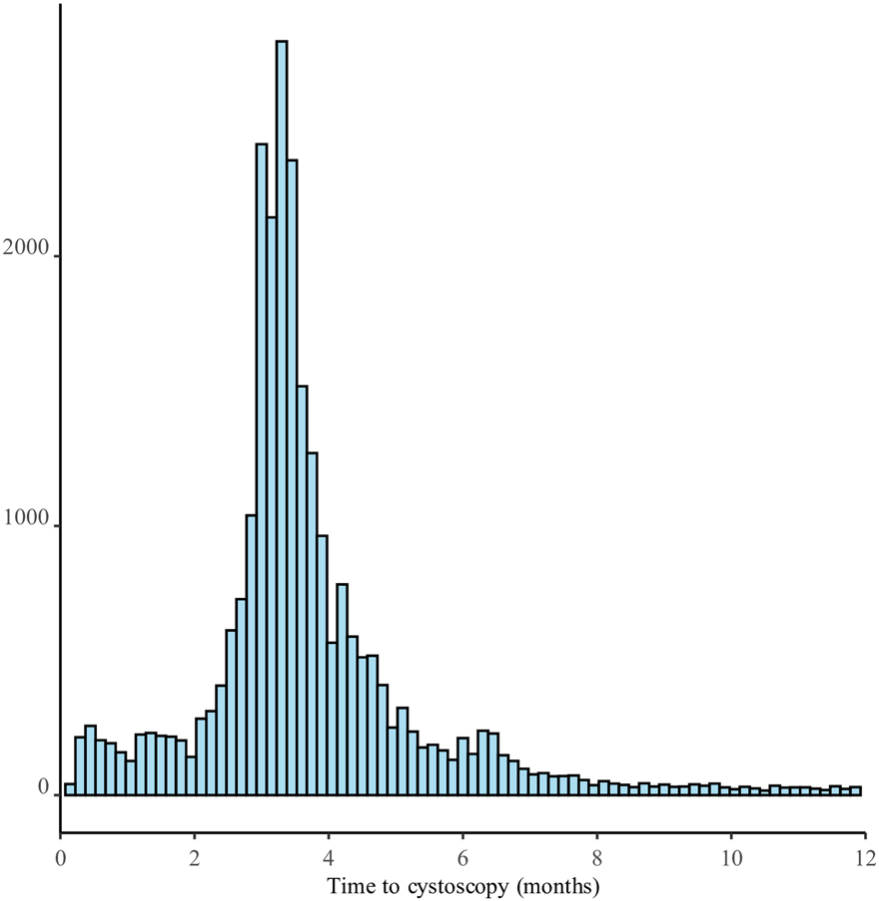
Distribution of time-to-first cystoscopy from the transurethral resection of bladder tumor.

In total, 26,660 newly diagnosed patients with NMIBC who underwent TUR-BT were included in this study: 2402 (9.0%) underwent their first cystoscopy within 2 months, 16,880 (63.3%) within 2–4 months, 4916 (18.4%) within 4–6 months, 1595 (6.0%) within 6–8 months, and 867 (3.3%) within 8–12 months. The median TTI was 3.17 (IQR, 0.94–3.79) months, and the mean TTI was 3.38 (SD, 1.72) (Fig. 2).

Table 1 summarizes the baseline demographic and clinical characteristics of patients. Across all TTI groups, male patients (21,653, 81.2%) exceeded the females (5007, 18.8%), with the highest numbers for both sexes occurring at a TTI of 2–4 months (males: 13,775, 81.6%; females: 3105, 18.4%; p = 0.006). Moreover, more patients were in the 65–74 age group than in the other age groups (p < 0.001). More patients were first diagnosed during 2013–2016 (10,432, 39.1%) than during 2004–2008 or 2009–2012. Patients with a CCI score of 2–3 accounted for the highest proportion across all TTI groups (p = 0.004).

**Table 1:** Baseline characteristics according to the time-to-first cystoscopy from the initial transurethral resection of bladder tumor.

Variable	Total (n = 26,660)	Time-to-treatment initiation					p-value
		0–2 mo (n = 2402)	2–4 mo (n = 16,880)	4–6 mo (n = 4916)	6–8 mo (n = 1595)	8–12 mo (n = 867)	
Sex, n (%)							0.006
Male	21,653 (81.2%)	1889 (78.6%)	13,775 (81.6%)	4007 (81.5%)	1273 (79.8%)	709 (81.8%)	
Female	5007 (18.8%)	513 (21.4%)	3105 (18.4%)	909 (18.5%)	322 (20.2%)	158 (18.2%)	
Age group at the NMIBC diagnosis year, n (%)							< 0.001
< 55	5107 (19.2%)	483 (20.1%)	3297 (19.5%)	826 (16.8%)	350 (21.9%)	151 (17.4%)	
55–64	6666 (25.0%)	633 (26.4%)	4253 (25.2%)	1190 (24.2%)	414 (26.0%)	176 (20.3%)	
65–74	8742 (32.8%)	761 (31.7%)	5510 (32.6%)	1724 (35.1%)	479 (30.0%)	268 (30.9%)	
≥ 75	6145 (23.0%)	525 (21.9%)	3820 (22.6%)	1176 (23.9%)	352 (22.1%)	272 (31.4%)	
NMIBC diagnosis year, n (%)							< 0.001
2004–2008	7781 (29.2%)	925 (38.5%)	4847 (28.7%)	1242 (25.3%)	478 (30.0%)	289 (33.3%)	
2009–2012	8447 (31.7%)	800 (33.3%)	5447 (32.3%)	1515 (30.8%)	423 (26.5%)	262 (30.2%)	
2013–2016	10,432 (39.1%)	677 (28.2%)	6586 (39.0%)	2159 (43.9%)	694 (43.5%)	316 (36.4%)	
Charlson comorbidity index score, n (%)	8394 (31.5%)	830 (34.6%)	5322 (31.5%)	1468 (29.9%)	507 (31.8%)	267 (30.8%)	0.004
0–1	8343 (31.3%)	735 (30.6%)	5319 (31.5%)	1528 (31.1%)	499 (31.3%)	262 (30.2%)	
2–3	9923 (37.2%)	837 (34.8%)	6239 (37.0%)	1920 (39.1%)	589 (36.9%)	338 (39.0%)	
≥ 4	4567 (17.1%)	368 (15.3%)	3003 (17.8%)	795 (16.2%)	261 (16.4%)	140 (16.1%)	
Repeated TUR-BT, n (%)	2074 (7.8%)	426 (17.7%)	620 (3.7%)	872 (17.7%)	123 (7.7%)	33 (3.8%)	< 0.001
BCG usage, n (%)	11,662 (43.7%)	788 (32.8%)	7980 (47.3%)	2327 (42.3%)	349 (21.9%)	218 (25.1%)	< 0.001
UTUC occurrence before the NMIBC diagnosis, n (%)	1128 (4.2%)	84 (3.5%)	731 (4.3%)	202 (4.1%)	73 (4.6%)	38 (4.4%)	0.366

*NMIBC* non-muscle invasive bladder cancer, *TUR-BT* transurethral resection of bladder tumor, *BCG* Bacillus Calmette-Guérin, *UTUC* upper urinary tract urothelial carcinoma, *mo* months.

Patients with TTIs of 0–2 and 4–6 months were more likely to undergo repeat TUR-BT (p < 0.001). Approximately 43.7% of all patients were administered BCG 2–6 weeks after the first TUR-BT, with the highest number of patients undergoing TTI at 2–4 months (p < 0.001). No significant difference was observed in TTI according to pre-diagnostic UTUC occurrence.

### The trends of risk for recurrence, progression, cancer-specific mortality, and all-cause mortality in patients with non-muscle invasive bladder cancer based on time-to-treatment initiation groups

**Figure 3:**
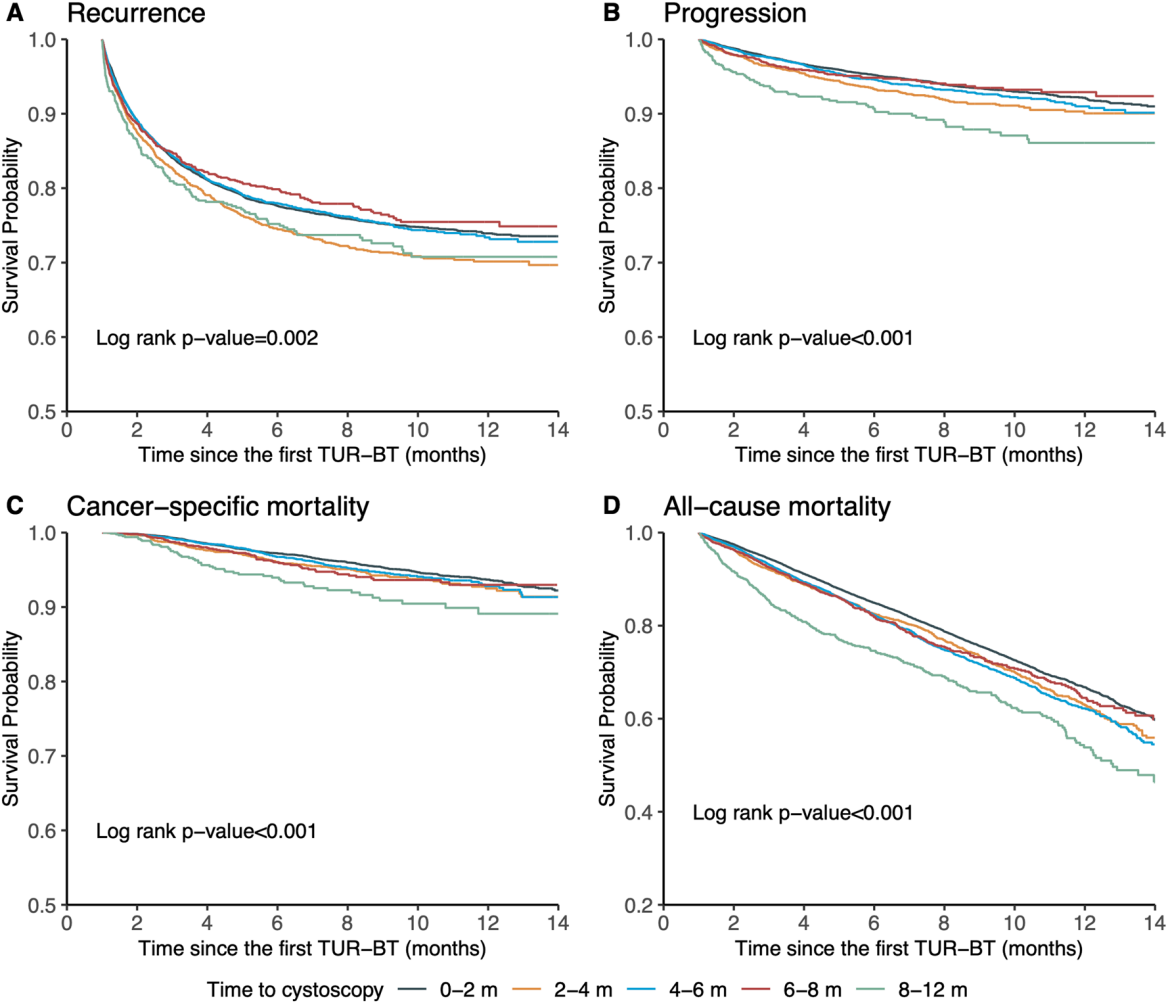
Kaplan–Meier survival curves for recurrence, progression, cancer-specific mortality, and all-cause mortality according to time to the first cystoscopy from the first transurethral resection of the bladder tumor.

Figure 3 illustrates the Kaplan–Meier curves for each outcome based on the TTIc. TTI groups within 0–2 and 8–12 months had lower survival probabilities of recurrence than those within the 2–4, 4–6, and 6–8 months. Patients with a TTI within 8–12 months had the lowest survival probability for all outcomes. Table 2 presents the adjusted hazard ratios (aHR) for each outcome in the TTI group. TTI within 0–2 months had a higher risk of progression (aHR 1.26; 95% confidence intervals [CI] 1.07–1.49; p = 0.007). Similarly, TTI within 8–12 months had a higher risk of progression (aHR 2.10; 95% CI 1.67–2.63; p < 0.001) and cancer-specific mortality (aHR 1.96; 95% CI 1.48–2.60; p < 0.001). However, no significant differences were observed in the risk of recurrence at any TTIc levels compared to TTI within 2–4 months. Progression and cancer-specific mortality did not significantly differ in the risk within 4–6 and 6–8 months compared with TTI within 2–4 months. The risk of mortality was significantly higher at all TTIc levels compared to the reference level of 2–4 months (Table 2).

**Table 2:** Hazard ratio for risk of recurrence, progression, cancer-specific mortality, and all-cause mortality according to time-to-first cystoscopy from the initial transurethral resection of bladder tumor.

Outcome	Adjusted hazard ratio (95% CI), p-value				
	0–2 months	2–4 months	4–6 months	6–8 months	8–12 months
Recurrence	1.08 (0.99–1.18)	1 (Ref)	1.00 (0.93–1.07)	0.92 (0.81–1.03)	1.09 (0.95–1.26)
	0.080		0.965	0.137	0.215
Progression	1.26 (1.07–1.49)	1 (Ref)	1.02 (0.88–1.17)	1.09 (0.86–1.37)	2.10 (1.67–2.63)
	0.007		0.797	0.474	< 0.001
Cancer-specific mortality	1.22 (0.99–1.49)	1 (Ref)	1.02 (0.86–1.22)	1.28 (0.98–1.66)	1.96 (1.48–2.60)
	0.062		0.817	0.067	< 0.001
All-cause mortality	1.18 (1.08–1.29)	1 (Ref)	1.11 (1.04–1.19)	1.16 (1.04–1.30)	1.52 (1.35–1.72)
	< 0.001		0.002	0.007	< 0.001

Adjusted for age, sex, diagnosis year, Charlson comorbidity index score, repeated TUR–BT, Bacillus Calmette-Guérin useage, and occurrence of upper urinary tract urothelial carcinoma.

*CI* confidence interval, *Ref* reference.

### Determination of the optimal timing of first cystoscopy after transurethral resection of bladder tumor for the risk of oncological outcomes

**Figure 4:**
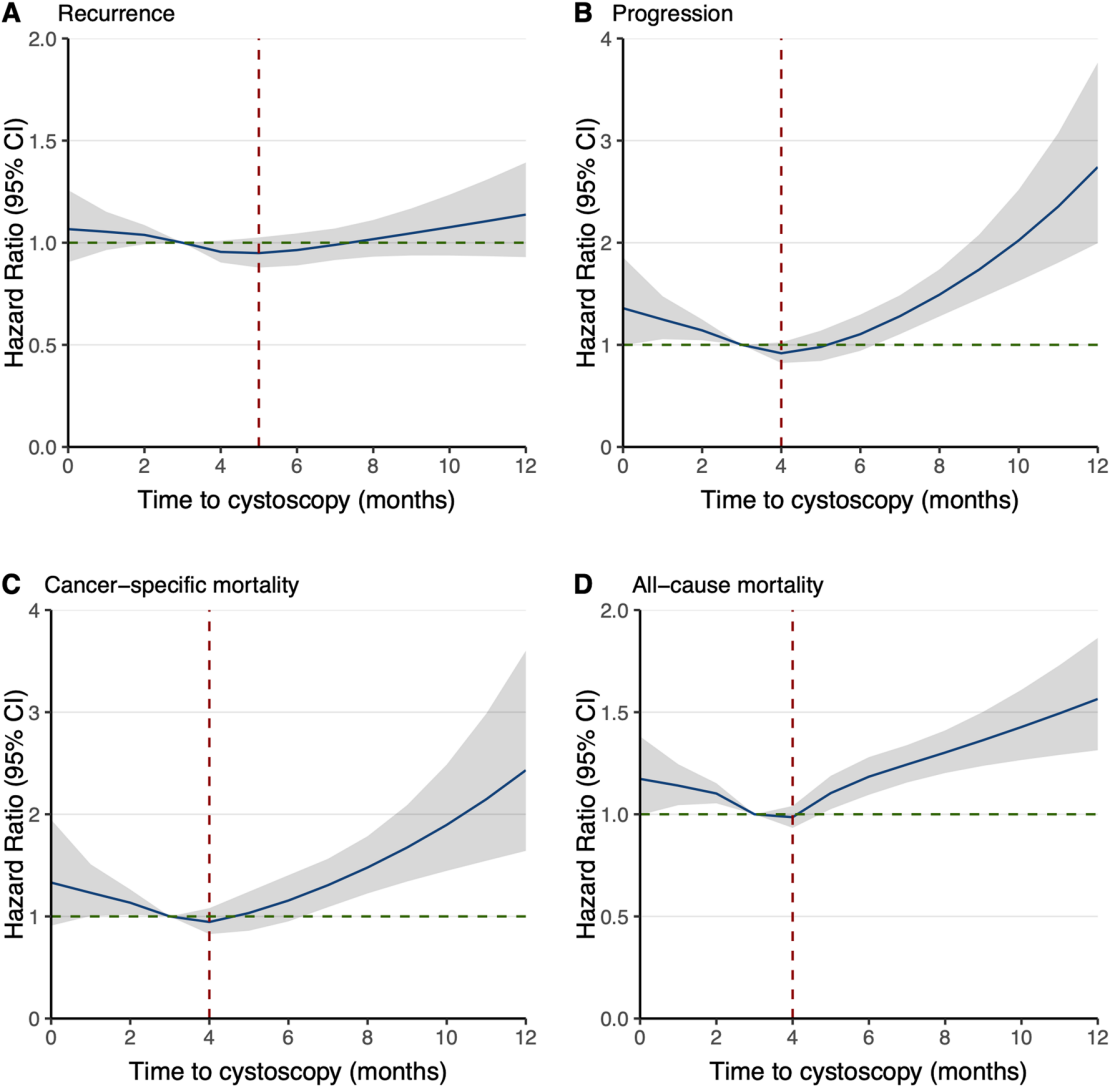
Associations between time-to-first cystoscopy from the first transurethral resection of bladder tumor and recurrence, progression, cancer-specific mortality, and all-cause mortality. Hazard ratios and 95% confidence intervals (CIs) were adjusted for age, sex, diagnosis year, CCI score, repeated TUR–BT, Bacillus Calmette-Guérin usage, and UTUC occurrence. Restricted cubic splines with knots at the 5th, 10th, 25th, 50th, 75th, 90th, and 95th percentiles of the time-to-cystoscopy distribution were used. The shaded areas indicate 95% pointwise CIs. The reference value for the hazard ratio was 3 months. The red dotted line represents the time of the lowest hazard ratios.

Restricted cubic spline analyses revealed a U-shaped trend in the association between TTI and the risks of all outcomes (Fig. 4). The lowest estimated hazard was at 5 months for recurrence (aHR 0.95; 95% CI 0.88–1.03; p = 0.187), whereas it was at 4 months for progression (aHR 0.92; 95% CI 0.82–1.02; p = 0.124), cancer-specific mortality (aHR 0.95; 95% CI 0.83–1.08; p = 0.409) and all-cause mortality (aHR 0.99; 95% CI 0.93–1.04; p = 0.603) compared to the reference TTI of 3 months. These findings were consistent with the trends in the TTI group analyses.

### Subgroup analyses

We conducted subgroup analyses according to age at NMIBC diagnosis, sex, CCI score, the repeated TUR-BT, and BCG usage after the first TUR-BT. Several analyses demonstrated a U-shaped trend between the TTI and the risks of all outcomes (Figs. S1, S2; Tables S1–S3). However, among patients aged < 55 years, the risk of recurrence and cancer-specific mortality decreased with increased time to first cystoscopy. For female patients, the risk of recurrence decreased over time to the first cystoscopy, which is similar for those with a CCI score ≥ 4. Among patients who underwent the repeated TUR-BT, the lowest estimated hazard ratios of progression and cancer-specific mortality were at 5 months. The BCG usage group had the lowest hazard ratios for recurrence, cancer-specific mortality, and all-cause mortality at 3 months.

## Discussion

Our study is the first to investigate the effects of cystoscopic TTI on four major outcomes: recurrence, progression, cancer-specific mortality, and all-cause mortality. TTI within 2–4 months was associated with the lowest all-cause mortality. Furthermore, TTI within 2–4 months had a lower risk of progression and cancer-specific mortality than TTI within 0–2 and 8–12 months. The lowest estimated hazard was at 4 months for progression, cancer-specific mortality and all-cause mortality. We demonstrated that the first follow-up cystoscopy could be related to the patient’s prognosis, and TTI at 4 months was favorable for the first cystoscopy. Thus, the optimal timing for the first cystoscopy follow-up at 4 months could affect oncologic prognosis in the clinical progression of patients with NMIBC. During the coronavirus disease pandemic in 2019, a 2–5-month delay in follow-up cystoscopy increased the risk of recurrence by 2.4-fold, and a > 3-month delay in cystoscopy increased the risk of progression by 6.7-fold^12^. Therefore, it is important to undergo cystoscopy at the appropriate time as planned.

First cystoscopy outcomes are critical prognostic factors, regardless of repeated TUR-BT or intravesical BCG usage^13^. The recurrence at three months revealed a relative risk of 4.0 for progression to muscle-invasive disease^8^. Identifying high-risk diseases and ensuring prompt treatment are fundamental to providing personalized care for NMIBC^14^. Early cystectomy of high-risk patients with NMIBC improved survival rates compared to delayed cystectomy after disease progression or resistant BCG therapy^14^. Hence, the timing of the first cystoscopy is highly heterogeneous in clinical practice. In this South Korean cohort study, only 63.3% of the patients underwent cystoscopy within 2–4 months. Standardization of follow-up timing is crucial to ensure appropriate subsequent treatment and prognosis prediction.

Most subgroup analyses revealed a U-shaped trend; however, the graph of females and those aged < 55 years depicted a linear graph in recurrence. In the female graph, a plausible explanation is the waiting-time paradox, which may be a contributing factor^15^. More aggressive tumors can be detected during early cystoscopy. The female sex increased the risk of recurrence, progression, and cancer-specific mortality in patients with NMIBC^16^. Despite a negative linear graph of recurrence in females, 3 or 4 months revealed the lowest estimated hazard ratio for progression and cancer-specific mortality. Patients aged < 55 years showed a different trend in progression and cancer-specific mortality compared to female patients. Both graphs did not rise steeply during the late periods, possibly because young patients have a relatively long life expectancy and account for a small proportion of the total cohort. Furthermore, younger age affected the outcomes of patients with NMIBC favorably^17^. Each patient’s characteristics can inspire precisional cystoscopy schedules.

Patients who underwent the repeated TUR-BT exhibited the lowest estimated hazard ratio for progression and cancer-specific mortality at five months, however, the risks did not significantly differ over time to cystoscopy. However, the risks of progression, cancer-specific mortality, and all-cause mortality were significantly lower at 3–4 months for those who did not undergo the repeated TUR-BT. In a meta-analysis, the repeated TUR-BT reduced the risk of recurrence, progression, cancer-specific mortality, and overall mortality^18,19^. Although the repeated TUR-BT could be related to high-risk patients or incomplete resection of initial TUR-BT, a similar U-shaped curve was shown regardless of the presence or absence of the repeated TUR-BT. The 3-month examination of BCG treatment was crucial for recurrence, cancer-specific mortality, and all-cause mortality; however, the difference was negligible compared with those who did not use BCG. Non-BCG users should undergo cystoscopy at 2–4 months because it differs from other TTIs.

Our study has some limitations. First, the major limitation is that the NHIS database did not provide information on the tumor stage or grade. Because this cohort does not contain a D code, very low-risk bladder cancer is likely to have been excluded. The BCG usage subgroup might not include low-risk NMIBC because intravesical BCG is recommended for intermediate or high-risk conditions^6^. Although we could not directly confirm the risk, we were able to estimate patients at high risk through the use of BCG. Second, this national database includes 99% Koreans; however, selection bias due to the retrospective nature cannot be avoided. The generalizability of the results may be limited to healthcare settings similar to that of the NHIS database; thus, their applicability to other settings or populations should be interpreted with caution. Third, unmeasured confounders, such as behavioral or environmental factors, may be observed. Fourth, not only the time to the first cystoscopy, but the interval for follow-up cystoscopy is also an important factor on oncological outcomes, which we did not consider in this study. It is of interest to identify optimal intervals for follow-up cystoscopy regarding oncologic outcomes in future research. Lastly, oncological outcomes defined by our operational definitions may differ from actual patient clinical outcomes. It is possible that the results of our study may have been underestimated, as some patients may have deteriorated before chemotherapy or radical cystectomy.

## Conclusions

Our study findings provide evidence for optimal first cystoscopy follow-up. The timing of the first cystoscopy follow-up was associated with oncologic prognosis. In our model, undergoing cystoscopy at 4 months has shown the best outcomes in clinical course. Therefore, patients who do not receive cystoscopy at approximately 4 months for any reason need more careful follow-up to predict a poor clinical course. And, if possible, the first cystoscopy at about 4 months after first TUR-BT is necessary to prevent a worse clinical course in primary NMIBC. However, for this study, which relies on real-world data and is retrospective in nature, further research is needed to provide a higher level of evidence.

### Supplementary Information

Supplementary Information.

## Data Availability

The data that support the findings of this study are available from the National Health Insurance Service but restrictions apply to the availability of these data, which were used under license for the current study, and so are not publicly available. Data are however available from the authors upon reasonable request and with permission of the National Health Insurance Service. Original raw data can be assessed through direct contact with the author, Se Young Choi (alse3025@gmail.com).
