# Supplementary material for: Optimal timing for the first cystoscopic follow-up using time-to-treatment initiation analysis of oncologic outcomes in primary non-muscle invasive bladder cancer
Source: Sci Rep. 2024 Apr 10;14:8440. doi: 10.1038/s41598-024-58809-x (PMC11006935; doi:10.1038/s41598-024-58809-x)
Supplement: Supplementary file 1 — Supplementary Information. [file 41598_2024_58809_MOESM1_ESM.docx]

Figure S1. Associations between time to the first cystoscopy from the first TUR-BT and recurrence, progression, cancer-specific mortality, and all-cause mortality according to age, sex CCI, the repeated TUR-BT, and BCG usage using the restricted cubic splines.

≥75

Figure S2. (Continued) Associations between time to the first cystoscopy from the first TUR-BT and recurrence, progression, cancer-specific mortality, and all-cause mortality according to age, sex, CCI, the repeated TUR-BT, and BCG usage using the restricted cubic splines.

≥4

Table S1. Hazard ratio for risk of recurrence, progression, cancer-specific mortality, and all-cause mortality according to time to the first cystoscopy from the first TUR-BT, stratified by age, sex, CCI, the repeated TUR-BT, and BCG usage.

| Age: < 55 (n = 5,107) | Adjusted Hazard Ratio (95% CI), p-value | | | | |
| --- | --- | --- | --- | --- | --- |
| Outcome | 0–2 m | 2–4 m | 4–6 m | 6–8 m | 8–12 m |
| Recurrence | 1.09 (0.88–1.35) | 1 (Ref) | 1.05 (0.88–1.25) | 0.59 (0.42–0.81) | 0.81 (0.54–1.22) |
|  | 0.420 |  | 0.611 | 0.001 | 0.307 |
| Progression | 1.98 (1.30–3.03) | 1 (Ref) | 1.44 (0.97–2.15) | 0.25 (0.06–1.04) | 2.01 (0.97–4.16) |
|  | 0.002 |  | 0.072 | 0.056 | 0.061 |
| Cancer-specific mortality | 2.26 (1.29–3.97) | 1 (Ref) | 1.61 (0.93–2.76) | - | 1.45 (0.45–4.70) |
|  | 0.004 |  | 0.087 | - | 0.533 |
| All-cause mortality | 1.56 (1.04–2.33) | 1 (Ref) | 1.44 (1.01–2.06) | 0.59 (0.28–1.27) | 2.55 (1.46–4.45) |
|  | 0.030 |  | 0.042 | 0.181 | 0.001 |
| Age: 55 – 64 (n=6,666) | Adjusted Hazard Ratio (95% CI), p-value | | | | |
| Outcome | 0–2 m | 2–4 m | 4–6 m | 6–8 m | 8–12 m |
| Recurrence | 1.05 (0.88–1.25) | 1 (Ref) | 1.02 (0.88–1.17) | 0.99 (0.80–1.24) | 1.18 (0.88–1.59) |
|  | 0.576 |  | 0.836 | 0.946 | 0.261 |
| Progression | 1.19 (0.88–1.6) | 1 (Ref) | 0.95 (0.73–1.24) | 0.83 (0.52–1.31) | 1.98 (1.27–3.10) |
|  | 0.259 |  | 0.729 | 0.417 | 0.003 |
| Cancer-specific mortality | 1.21 (0.84–1.75) | 1 (Ref) | 0.87 (0.61–1.23) | 0.97 (0.57–1.66) | 1.52 (0.82–2.80) |
|  | 0.313 |  | 0.428 | 0.919 | 0.183 |
| All-cause mortality | 1.35 (1.08–1.69) | 1 (Ref) | 1.08 (0.88–1.32) | 1.02 (0.74–1.42) | 1.44 (0.97–2.13) |
|  | 0.009 |  | 0.478 | 0.891 | 0.067 |
| Age: 65 – 74 (n=8,742) | Adjusted Hazard Ratio (95% CI), p-value | | | | |
| Outcome | 0–2 m | 2–4 m | 4–6 m | 6–8 m | 8–12 m |
| Recurrence | 1.13 (0.97–1.30) | 1 (Ref) | 0.95 (0.85–1.08) | 0.97 (0.80–1.17) | 1.09 (0.85–1.38) |
|  | 0.112 |  | 0.442 | 0.732 | 0.507 |
| Progression | 1.19 (0.91–1.56) | 1 (Ref) | 0.97 (0.77–1.2) | 1.49 (1.08–2.04) | 2.30 (1.64–3.22) |
|  | 0.200 |  | 0.760 | 0.014 | <0.001 |
| Cancer-specific mortality | 1.09 (0.79–1.51) | 1 (Ref) | 0.99 (0.76–1.29) | 1.59 (1.10–2.28) | 2.24 (1.50–3.34) |
|  | 0.606 |  | 0.957 | 0.013 | <0.001 |
| All-cause mortality | 1.17 (1.01–1.35) | 1 (Ref) | 1.15 (1.02–1.29) | 1.21 (1.01–1.45) | 1.46 (1.18–1.81) |
|  | 0.032 |  | 0.018 | 0.039 | <0.001 |
| Age: ≥75 (n=6,145) | Adjusted Hazard Ratio (95% CI), p-value | | | | |
| Outcome | 0–2 m | 2–4 m | 4–6 m | 6–8 m | 8–12 m |
| Recurrence | 1.04 (0.85–1.27) | 1 (Ref) | 1.02 (0.88–1.18) | 1.05 (0.83–1.34) | 1.19 (0.91–1.56) |
|  | 0.696 |  | 0.809 | 0.681 | 0.198 |
| Progression | 1.01 (0.63–1.62) | 1 (Ref) | 1.00 (0.71–1.41) | 1.23 (0.72–2.11) | 1.75 (1.02–3.00) |
|  | 0.969 |  | 0.982 | 0.445 | 0.041 |
| Cancer-specific mortality | 0.95 (0.55–1.64) | 1 (Ref) | 1.04 (0.71–1.53) | 1.62 (0.94–2.79) | 2.02 (1.13–3.62) |
|  | 0.856 |  | 0.844 | 0.084 | 0.018 |
| All-cause mortality | 1.11 (0.98–1.26) | 1 (Ref) | 1.07 (0.97–1.18) | 1.21 (1.04–1.41) | 1.49 (1.26–1.76) |
|  | 0.101 |  | 0.178 | 0.017 | <0.001 |

Table S2. (continued) Hazard ratio for risk of recurrence, progression, cancer-specific mortality, and all-cause mortality according to time to the first cystoscopy from the first TUR-BT, stratified by age, sex, CCI, the repeated TUR-BT, and BCG usage.

| Male (n = 21,653) | Adjusted Hazard Ratio (95% CI), p-value | | | | |
| --- | --- | --- | --- | --- | --- |
| Outcome | 0–2 m | 2–4 m | 4–6 m | 6–8 m | 8–12 m |
| Recurrence | 1.06 (0.96–1.17) | 1 (Ref) | 1.03 (0.95–1.11) | 0.97 (0.85–1.10) | 1.18 (1.02–1.38) |
|  | 0.289 |  | 0.525 | 0.582 | 0.029 |
| Progression | 1.19 (0.99–1.43) | 1 (Ref) | 1.01 (0.87–1.17) | 1.10 (0.86–1.40) | 2.00 (1.56–2.56) |
|  | 0.066 |  | 0.918 | 0.468 | <0.001 |
| Cancer-specific mortality | 1.23 (0.99–1.53) | 1 (Ref) | 0.99 (0.82–1.20) | 1.33 (1.01–1.75) | 1.87 (1.38–2.53) |
|  | 0.066 |  | 0.932 | 0.045 | <0.001 |
| All-cause mortality | 1.19 (1.09–1.31) | 1 (Ref) | 1.11 (1.03–1.20) | 1.22 (1.08–1.37) | 1.45 (1.27–1.66) |
|  | <0.001 |  | 0.005 | 0.001 | <0.001 |
| Female (n = 5,007) | Adjusted Hazard Ratio (95% CI), p-value | | | | |
| Outcome | 0–2 m | 2–4 m | 4–6 m | 6–8 m | 8–12 m |
| Recurrence | 1.20 (0.99–1.45) | 1 (Ref) | 0.87 (0.72–1.04) | 0.70 (0.52–0.94) | 0.67 (0.43–1.03) |
|  | 0.069 |  | 0.116 | 0.018 | 0.071 |
| Progression | 1.67 (1.12–2.51) | 1 (Ref) | 1.08 (0.73–1.61) | 0.97 (0.52–1.81) | 2.87 (1.57–5.24) |
|  | 0.013 |  | 0.699 | 0.916 | <0.001 |
| Cancer-specific mortality | 1.10 (0.62–1.95) | 1 (Ref) | 1.23 (0.77–1.98) | 0.88 (0.40–1.93) | 2.80 (1.34–5.87) |
|  | 0.738 |  | 0.383 | 0.747 | 0.006 |
| All-cause mortality | 1.12 (0.91–1.38) | 1 (Ref) | 1.11 (0.94–1.32) | 0.92 (0.69–1.23) | 2.00 (1.5–2.66) |
|  | 0.276 |  | 0.226 | 0.573 | <0.001 |
|  |  |  |  |  |  |
|  |  |  |  |  |  |
| CCI: 0 – 1 (n = 8,394) | Adjusted Hazard Ratio (95% CI), p-value | | | | |
| Outcome | 0–2 m | 2–4 m | 4–6 m | 6–8 m | 8–12 m |
| Recurrence | 1.06 (0.91–1.23) | 1 (Ref) | 1.10 (0.98–1.25) | 0.90 (0.73–1.10) | 1.31 (1.04–1.65) |
|  | 0.466 |  | 0.109 | 0.298 | 0.024 |
| Progression | 1.10 (0.82–1.46) | 1 (Ref) | 1.02 (0.80–1.3) | 1.19 (0.82–1.72) | 2.47 (1.73–3.54) |
|  | 0.527 |  | 0.886 | 0.371 | <0.001 |
| Cancer-specific mortality | 1.11 (0.78–1.59) | 1 (Ref) | 1.05 (0.78–1.42) | 1.45 (0.95–2.22) | 2.22 (1.42–3.47) |
|  | 0.546 |  | 0.741 | 0.082 | <0.001 |
| All-cause mortality | 1.17 (1.00–1.37) | 1 (Ref) | 1.14 (1.00–1.31) | 1.07 (0.86–1.34) | 1.31 (1.03–1.66) |
|  | 0.054 |  | 0.049 | 0.546 | 0.025 |
| CCI: 2 – 3 (n = 8,343) | Adjusted Hazard Ratio (95% CI), p-value | | | | |
| Outcome | 0–2 m | 2–4 m | 4–6 m | 6–8 m | 8–12 m |
| Recurrence | 1.06 (0.90–1.24) | 1 (Ref) | 0.99 (0.87–1.12) | 0.91 (0.74–1.12) | 1.12 (0.87–1.44) |
|  | 0.501 |  | 0.832 | 0.357 | 0.383 |
| Progression | 1.42 (1.07–1.87) | 1 (Ref) | 1.07 (0.84–1.37) | 0.67 (0.40–1.11) | 2.01 (1.33–3.04) |
|  | 0.015 |  | 0.577 | 0.122 | <0.001 |
| Cancer-specific mortality | 1.37 (0.97–1.92) | 1 (Ref) | 1.11 (0.82–1.50) | 0.72 (0.40–1.30) | 2.09 (1.30–3.36) |
|  | 0.072 |  | 0.495 | 0.276 | 0.002 |
| All-cause mortality | 1.25 (1.07–1.46) | 1 (Ref) | 1.13 (0.99–1.28) | 1.14 (0.93–1.39) | 1.57 (1.26–1.95) |
|  | 0.004 |  | 0.072 | 0.203 | <0.001 |
| CCI: ≥ 4 (n = 9,923) | Adjusted Hazard Ratio (95% CI), p-value | | | | |
| Outcome | 0–2 m | 2–4 m | 4–6 m | 6–8 m | 8–12 m |
| Recurrence | 1.13 (0.98–1.32) | 1 (Ref) | 0.92 (0.82–1.04) | 0.94 (0.78–1.15) | 0.88 (0.68–1.15) |
|  | 0.099 |  | 0.172 | 0.553 | 0.341 |
| Progression | 1.31 (0.96–1.77) | 1 (Ref) | 0.97 (0.76–1.24) | 1.4 (0.98–2.00) | 1.75 (1.14–2.68) |
|  | 0.087 |  | 0.81 | 0.067 | 0.01 |
| Cancer-specific mortality | 1.19 (0.81–1.75) | 1 (Ref) | 0.91 (0.67–1.24) | 1.66 (1.10–2.48) | 1.50 (0.86–2.64) |
|  | 0.369 |  | 0.549 | 0.015 | 0.156 |
| All-cause mortality | 1.14 (1.00–1.30) | 1 (Ref) | 1.09 (0.98–1.21) | 1.23 (1.05–1.45) | 1.64 (1.36–1.97) |
|  | 0.056 |  | 0.106 | 0.013 | <0.001 |

Table S3. (Continued) Hazard ratio for risk of recurrence, progression, cancer-specific mortality, and all-cause mortality according to time to the first cystoscopy from the first TUR-BT, stratified by age, sex, CCI, the repeated TUR-BT, and BCG usage.

| Repeated TUR-BT: No  (n = 24,586) | Adjusted Hazard Ratio (95% CI), p-value | | | | |
| --- | --- | --- | --- | --- | --- |
| Outcome | 0–2 m | 2–4 m | 4–6 m | 6–8 m | 8–12 m |
| Recurrence | 1.07 (0.98–1.18) | 1 (Ref) | 0.99 (0.91–1.06) | 0.92 (0.81–1.03) | 1.06 (0.91–1.23) |
|  | 0.151 |  | 0.715 | 0.153 | 0.443 |
| Progression | 1.27 (1.05–1.53) | 1 (Ref) | 1.05 (0.90–1.23) | 1.17 (0.92–1.48) | 2.08 (1.64–2.63) |
|  | 0.012 |  | 0.501 | 0.203 | <0.001 |
| Cancer-specific mortality | 1.24 (0.99–1.55) | 1 (Ref) | 1.06 (0.88–1.28) | 1.34 (1.03–1.75) | 1.93 (1.45–2.59) |
|  | 0.057 |  | 0.538 | 0.031 | <0.001 |
| All-cause mortality | 1.16 (1.05–1.27) | 1 (Ref) | 1.15 (1.07–1.23) | 1.16 (1.04–1.30) | 1.52 (1.34–1.72) |
|  | 0.002 |  | <0.001 | 0.008 | <0.001 |
| Repeated TUR-BT: Yes  (n = 2,074) | Adjusted Hazard Ratio (95% CI), p-value | | | | |
| Outcome | 0–2 m | 2–4 m | 4–6 m | 6–8 m | 8–12 m |
| Recurrence | 1.27 (0.96–1.67) | 1 (Ref) | 1.23 (0.97–1.57) | 1.02 (0.62–1.67) | 2.29 (1.26–4.17) |
|  | 0.091 |  | 0.089 | 0.938 | 0.007 |
| Progression | 1.03 (0.67–1.58) | 1 (Ref) | 0.72 (0.48–1.07) | 0.44 (0.16–1.23) | 2.45 (1.04–5.77) |
|  | 0.889 |  | 0.100 | 0.117 | 0.041 |
| Cancer-specific mortality | 0.87 (0.50–1.54) | 1 (Ref) | 0.64 (0.38–1.08) | 0.60 (0.18–1.98) | 2.26 (0.79–6.49) |
|  | 0.644 |  | 0.094 | 0.401 | 0.131 |
| All-cause mortality | 1.17 (0.90–1.53) | 1 (Ref) | 0.82 (0.63–1.06) | 1.03 (0.63–1.69) | 1.58 (0.82–3.05) |
|  | 0.250 |  | 0.133 | 0.891 | 0.168 |
|  |  |  |  |  |  |
|  |  |  |  |  |  |
| BCG Usage: No (n = 14,998) | Adjusted Hazard Ratio (95% CI), p-value | | | | |
| Outcome | 0–2 m | 2–4 m | 4–6 m | 6–8 m | 8–12 m |
| Recurrence | 1.07 (0.96–1.19) | 1 (Ref) | 0.99 (0.90–1.10) | 0.84 (0.73–0.96) | 1.09 (0.93–1.29) |
|  | 0.253 |  | 0.916 | 0.012 | 0.280 |
| Progression | 1.59 (1.28–1.96) | 1 (Ref) | 1.17 (0.95–1.43) | 1.12 (0.85–1.49) | 2.48 (1.89–3.25) |
|  | <0.001 |  | 0.142 | 0.427 | <0.001 |
| Cancer-specific mortality | 1.48 (1.14–1.91) | 1 (Ref) | 1.02 (0.79–1.32) | 1.20 (0.87–1.66) | 2.35 (1.70–3.25) |
|  | 0.003 |  | 0.880 | 0.266 | <0.001 |
| All-cause mortality | 1.29 (1.16–1.43) | 1 (Ref) | 1.17 (1.06–1.28) | 1.14 (1.00–1.29) | 1.69 (1.47–1.94) |
|  | <0.001 |  | 0.002 | 0.053 | <0.001 |
| BCG Usage: Yes (n = 11,662) | Adjusted Hazard Ratio (95% CI), p-value | | | | |
| Outcome | 0–2 m | 2–4 m | 4–6 m | 6–8 m | 8–12 m |
| Recurrence | 1.11 (0.95–1.29) | 1 (Ref) | 1.01 (0.90–1.12) | 1.23 (0.98–1.53) | 1.08 (0.80–1.45) |
|  | 0.173 |  | 0.906 | 0.077 | 0.626 |
| Progression | 0.9 (0.68–1.21) | 1 (Ref) | 0.89 (0.73–1.09) | 1.15 (0.77–1.73) | 1.59 (1.03–2.47) |
|  | 0.496 |  | 0.269 | 0.483 | 0.037 |
| Cancer-specific mortality | 0.88 (0.61–1.25) | 1 (Ref) | 1.02 (0.80–1.29) | 1.54 (0.99–2.40) | 1.29 (0.70–2.35) |
|  | 0.467 |  | 0.900 | 0.055 | 0.412 |
| All-cause mortality | 1.03 (0.89–1.18) | 1 (Ref) | 1.07 (0.97–1.18) | 1.30 (1.05–1.59) | 1.09 (0.83–1.44) |
|  | 0.708 |  | 0.170 | 0.014 | 0.522 |

Adjusted for age, sex, diagnosis year, Charlson comorbidity index score, repeated TUR–BT, Bacillus Calmette-Guérin useage, and occurrence of upper urinary tract urothelial carcinoma

CI, confidence interval; Ref, reference
